# Supplementary material for: Close social relationships correlate with human gut microbiota composition
Source: Sci Rep. 2019 Jan 24;9:703. doi: 10.1038/s41598-018-37298-9 (PMC6345772; doi:10.1038/s41598-018-37298-9)
Supplement: Supplementary file 1 — Supplementary Materials [file 41598_2018_37298_MOESM1_ESM.docx]

**SUPPLEMENTARY MATERIALS**

**Close social relationships correlate with human gut microbiota composition**

Kimberly A. Dill-McFarland^1,2†#^, Zheng-Zheng Tang^3,4#^, Julia H. Kemis^1^, Robert L. Kerby^1^, Guanhua Chen^3^, Alberto Palloni^5^, Thomas Sorenson^1^, Federico E. Rey^1*^, Pamela Herd^2,5*^

^1^Department of Bacteriology, U. of Wisconsin-Madison, 1550 Linden Drive, Madison, WI, USA 53706;

^2^Center for the Demography of Health and Aging, 1180 Observatory Drive, Madison, WI, USA, 53706;

^3^Department of Biostatistics and Medical Informatics, U. of Wisconsin-Madison, 600 Highland Avenue, Madison, WI, USA, 53792

^4^Wisconsin Institute for Discovery, 330 N Orchard St, Madison, WI, USA, 53715

^5^Department of Sociology, U. of Wisconsin-Madison, 1180 Observatory Drive, Madison, WI, USA, 53706

^†^Current address: Department of Microbiology and Immunology, U. of British Columbia, 2350 Health Sciences Mall, Vancouver, BC, Canada, V6T 1Z3

# Equal contribution

**Figure S1.** Factors associated with overall fecal microbiota. Non-metric multidimensional scaling (nMDS) of beta-diversity for all graduates. (A) Bray-Curtis, (B) Jaccard, and (C) weighted UniFrac metrics are shown. Variables found to be significant (PERMANOVA P < 0.05, red) and trends (0.05 < P < 0.1, black) are shown as fitted arrows. Arrows point toward increasing values (dietary protein), toward affirmative responses (high blood sugar, antibiotics, heart disease), or from male to female (sex).


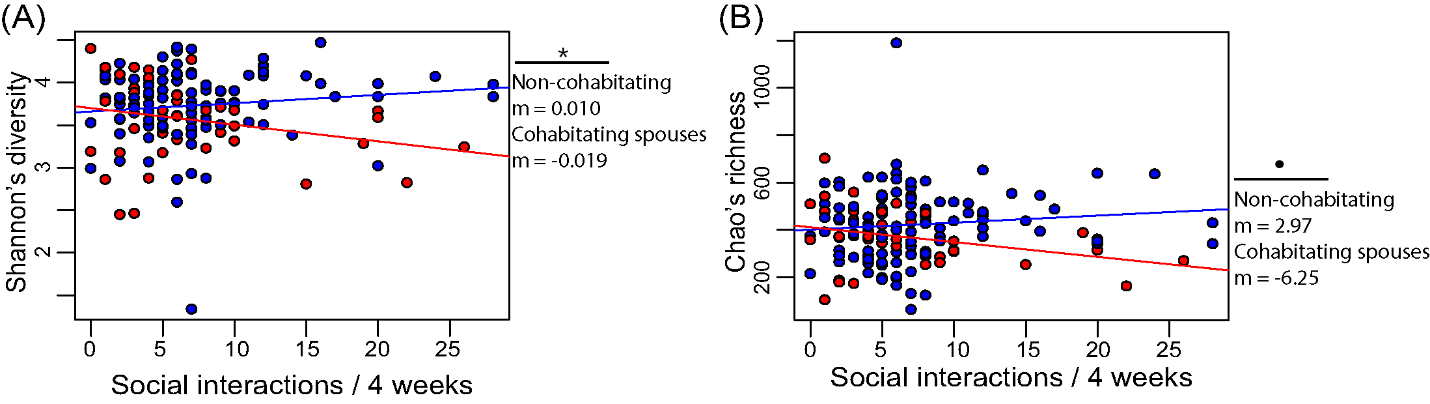


**Figure S2.** The relationship between socialness and alpha-diversity differ among cohabitating spouses and non-cohabitating individuals. Linear models of (A) Shannon’s diversity and (B) Chao’s richness by the interaction of socialness and cohabitation. Slopes (m) were different for cohabitating (red) and non-cohabiting (blue) groups but not significantly different from zero within each group. *P < 0.05, •P < 0.1


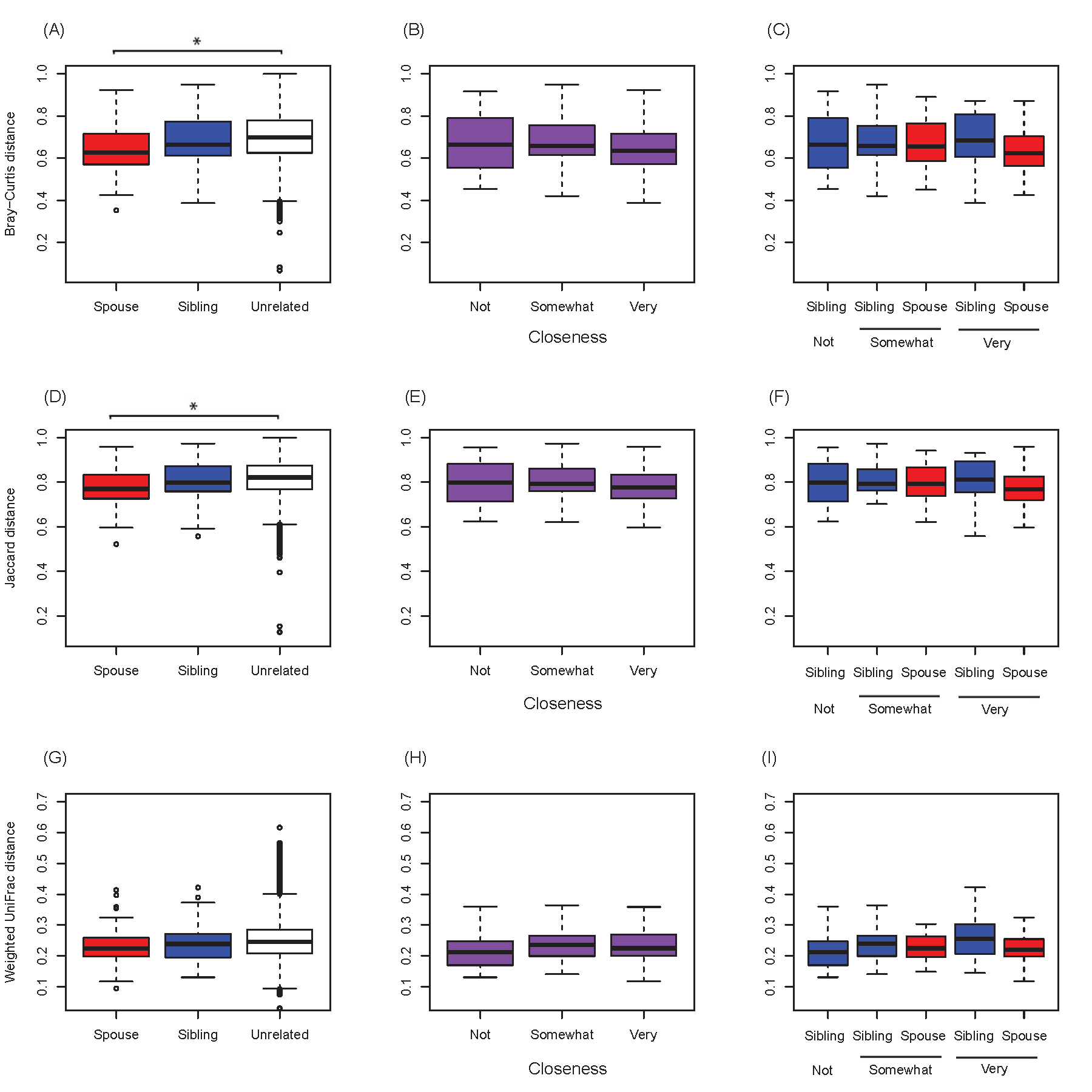


**Figure S3.** Microbial sharing in spouse and sibling relationships. Beta-diversity metrics (A,B,C) Bray-Curtis, (D,E,F) Jaccard, and (G,H,I) weighted UniFrac were tested. Groups were (A,D,G) spouse, sibling, and unrelated pairs, (B,E,H) spouses and siblings grouped by relationship closeness, and (C,F,I) spouses and siblings separated by relationship closeness. Statistical tests were performed for comparing different groups (using the same approach as that for Figure 3). Closeness was not significant for these metrics. *P < 0.05

**Table S1.** Differences in beta-diversity across graduates. P-values are reported for PERMANOVA and Mantel tests..

|  | Microbial beta-diversity metric | | | |
| --- | --- | --- | --- | --- |
| Comparison | Bray-Curtis | Jaccard | Weighted UniFrac | Unweighted UniFrac |
| Age | < 0.001 | < 0.001 | < 0.001 | < 0.001 |
| Sex | < 0.001 | < 0.001 | < 0.001 | < 0.001 |
| Antibiotics | 0.004 | 0.010 | 0.002 | 0.004 |
| Dietary protein | 0.012 | 0.006 | 0.006 | 0.063 |
| Dietary beta-diversity, Bray-Curtis | 0.142 | 0.048 | 0.132 | 0.154 |
| Dietary beta-diversity, Jaccard | 0.010 | 0.007 | 0.005 | 0.058 |
| Diabetes | 0.001 | 0.002 | 0.003 | 0.003 |
| Heart disease | 0.060 | 0.107 | 0.087 | 0.055 |
| Social interaction : cohabitation | 4.2e-03 | 3.2e-03 | 7.8e-02 | 3.0e-03 |

**Table S2.** Differences in beta-diversity across graduates, spouses, siblings, and unrelated individuals. The regression p-values and parameter estimates (in the parentheses) are reported. The group at the left is coded as “1” and at the right is coded as “0”. *

|  |  |  |  |  | Microbial beta-diversity metric | | | |  |
| --- | --- | --- | --- | --- | --- | --- | --- | --- | --- |
| Comparison | | | | Cofounder | Bray-Curtis | Jaccard | Weighted UniFrac | Unweighted UniFrac | |
| Spouse | vs. |  | Sibling | Age & Sex | 0.24  (-0.029) | 0.27  (-0.020) | 0.36  (-0.011) | 1.3e-02  (-0.039) | |
|  |  |  |  | All | 0.33  (-0.025) | 0.37  (-0.017) | 0.35  (-0.012) | 3.3e-02  (-0.036) | |
| Spouse | vs. |  | Unrelated | Age & Sex | 1.2e-03  (-0.065) | 1.1e-03  (-0.048) | 2.8e-02  (-0.023) | 7.8e-06  (-0.060) | |
|  |  |  |  | All | 2.4e-03  (-0.064) | 2.3e-03  (-0.046) | 3.4e-02  (-0.023) | 3.2e-05  (-0.058) | |
| Sibling | vs. |  | Unrelated | Age & Sex | 0.24  (-0.026) | 0.20  (-0.020) | 0.42  (-0.0087) | 0.34  (-0.013) | |
|  |  |  |  | All | 0.31  (-0.027) | 0.27  (-0.021) | 0.49  (-0.0075) | 0.39  (-0.012) | |

* Comparisons are at the pair level where beta-diversity is used as the outcome in the regression analysis, allowing for effect estimates. The reference group (coded as 0) is at always at column 2 and the alternative group (coded as 1) is at column 1. Negative estimate means the group on the left has smaller diversity (i.e. more similar) than the group on the right.

**Table S3.** Differences in beta-diversity across spouses, siblings, and unrelated individuals by relationship closeness. The regression p-values and parameter estimates (in the parentheses) are reported. The group at the left is coded as “1” and at the right is coded as “0”. *

|  |  |  |  | Microbial beta-diversity metric | | | |
| --- | --- | --- | --- | --- | --- | --- | --- |
| Comparison | | | Cofounder | Bray-Curtis | Jaccard | Weighted UniFrac | Unweighted UniFrac |
| Very close spouse | vs. | Unrelated | Age & Sex | 1.6e-03  (-0.073) | 1.5e-03  (-0.053) | 3.7e-02  (-0.025) | 2.4e-05  (-0.065) |
|  |  |  | All | 3.3e-03  (-0.071) | 3.1e-03  (-0.052) | 4.9e-02  (-0.025) | 5.2e-05  (-0.065) |
| Somewhat close spouse | vs. | Unrelated | Age & Sex | 0.39  (-0.037) | 0.38  (-0.026) | 0.44  (-0.017) | 0.20  (-0.039) |
|  |  |  | All | 0.39  (-0.037) | 0.39  (-0.026) | 0.41  (-0.019) | 0.24  (-0.037) |
| Very close sibling | vs. | Unrelated | Age & Sex | 0.56  (-0.022) | 0.52  (-0.019) | 0.60  (0.0059) | 0.39  (-0.02) |
|  |  |  | All | 0.51  (-0.032) | 0.49  (-0.026) | 0.60  (0.0048) | 0.36  (-0.026) |
| Somewhat close sibling | vs. | Unrelated | Age & Sex | 0.43  (-0.020) | 0.42  (-0.014) | 0.44  (-0.0098) | 0.51  (-0.0087) |
|  |  |  | All | 0.46  (-0.022) | 0.45  (-0.015) | 0.48  (-0.0086) | 0.55  (-0.0080) |
| Not close sibling | vs. | Unrelated | Age & Sex | 0.33  (-0.053) | 0.29  (-0.041) | 0.33  (-0.029) | 0.51  (-0.016) |
|  |  |  | All | 0.38  (-0.058) | 0.36  (-0.044) | 0.42  (-0.031) | 0.47  (-0.021) |

* Comparisons are at the pair level where beta-diversity is used as the outcome in the regression analysis, allowing for effect estimates. The reference group (coded as 0) is at always at column 2 and the alternative group (coded as 1) is at column 1. Negative estimate means the group on the left has smaller diversity (i.e. more similar) than the group on the right.

Table S.4 ANOVA of diet beta-diversity metrics against relationship closeness.

|  | All | Spouses | Siblings |
| --- | --- | --- | --- |
| Bray-Curtis protein* | 0.533 | 0.616 | 0.586 |
| Bray-Curtis overall diet** | 0.781 | 0.745 | 0.802 |
| Jaccard overall diet*** | 0.973 | 0.621 | 0.818 |

Note:

*Bray-Curtis of protein consumption expressed as times per week for 4 items

**Bray-Curtis of overall diet expressed as percent of individual dietary components consumed regularly. Protein (4), vegetables (76), fruit (24). Using percentages corrects for differential representation of each category (e.g. 4 vs. 76 vs. 24 items)

***Jaccard of overall diet expressed as Y/N regular consumption of the same protein, vegetable, and fruit items as in Bray-Curtis of overall diet consumption but retained as individual items in the table.

**Text S1.** Details of WLS survey questions used in this study.

Dietary consumption is based on a Food Frequency Questionnaire approach that captures habitual diet. We follow common practice as these are employed in most larger studies, including recent microbiome studies, to measure nutrition. The approach has been shown to be valid and reliable, including in older adults; it is correlated with more immediate food recall measures (Horwath, C. C. "Validity of a short food frequency questionnaire for estimating nutrient intake in elderly people." *British Journal of Nutrition* 70, no. 1 (1993): 3-14.).

The survey was administered at time of fecal sampling (see below regarding question wording for nutrition items).

iq Best measure of IQ mapped from converted Henmon-Nelson score

kinship ¼ k1 + ½ k2 ^75^

k1 = probability that one allele is identical by descent (IBD)

k2 = probability that both alleles are IBD

94,261 selected single nucleotide polymorphisms (SNPs) from Infinium OmniExpress BeadChip (Illumina, San Diego, CA)

- Autosomal
- Minor allele frequency (MAF) > 5%
- Missing call rate < 2%
- Linkage disequilibrium (LD) pruning using sliding 10 Mb window with r^2^ threshold of 0.1

Participants were asked: “The next set of questions asks about the kind of things you eat such as meat, vegetables, and dairy products. Now thinking about the kinds of things you at in the past year…on how many days did you eat [protein/meat categories listed below] during a typical week?”

q13 Frequency of red meat, 0-7 days a week

q14 Frequency of poultry, 0-7 days a week

q15 Frequency of pork, 0-7 days a week

q16 Frequency of seafood, 0-7 days a week

q17_ “Think about the vegetables you at regularly during the past year. By regularly, we mean you ate that vegetable at least once per week, either year-round OR when it was in season. Using the list below, please check the box for any vegetable you ate regularly during the past year.”

| 1 alfalfa sprouts  2 artichoke hearts  3 artichokes  4 asparagus  5 avocado  6 baking potatoes  7 bamboo shoots  8 bean sprouts  9 beet greens  10 beets  11 bitter melon  12 bok choy  13 broccoli  14 brussel sprouts  15 green cabbage  16 carrots  17 cauliflower  18 celery  19 celery root  20 chard  21 corn  22 cucumbers  23 daikon  24 dandelion greens  25 edamame  26 eggplant  27 endive | 28 escarole  29 fennel  30 frisee  31 garlic  32 green beans  33 green leaf lettuce  34 green onions  35 peppers  36 jicama  37 kale  38 kohlrabi  39 leeks  40 lotus root  41 mustard greens  42 napa  43 okra  44 onions  45 orange peppers  46 palm hearts  47 parsnips  48 patty pan squash  49 pea shoots  50 peas  51 poblano chiles  52 mushrooms  53 potatoes  54 pumpkin | 55 purple beans  56 radicchio  57 radishes  58 lettuce  59 rutabagas  60 sauerkraut  61 seaweed  62 shallots  63 snow peas  64 spinach  65 squash (any kind)  66 sugar snap peas  67 sweet potatoes  68 taro root  69 tomatoes  70 turnips  71 water chestnuts  72 wax beans  73 yucca  74 zucchini  75 any fermented vegetable  76 other  77 did not eat any vegetables regularly |
| --- | --- | --- |

q18_ Fruit eaten regularly, yes or no

| 1 apple  2 apricot  3 banana  4 berries (any kind)  5 cantaloupe  6 cherry  7 clementine/ tangerine  8 honeydew | 9 kiwi  10 kumquat  11 lemon  12 lime  13 mango  14 nectarine  15 orange  16 papaya  17 peach | 18 pear  19 pineapple  20 pomegranate  21 starfruit  22 ugli  23 watermelon  24 other  25 did not eat any fruit regularly |
| --- | --- | --- |

q20 Currently own a pet, yes or no

q21_a Pets in home; dogs

q21_b Pets in home; cats

q21_c Pets in home; birds

q21_d Pets in home; reptiles

q21_e Pets in home; other

q24 Antibiotics in the last six months, yes or no

WLS: Graduates

sexrsp Sex of graduate respondent.

brdxdy Graduate's year of birth.

OCF357 Was Father's 1957 occupation farming?

hf017j1e 1990 Major Occupation Code for first or only job in Participant's first employer job spell.

hb103red Summary of equivalent years of regular education based on highest degree.

hc101re Are you living in a marriage-like relationship or cohabiting?

HD01701-06 Does the [1-6] child live with the Participant?

ha103re Interviewer's assessment of participant's grooming.

jz023rer How many times, if at all, during the past four weeks have you gotten together with friends?

jz024rer How many times, if at all, during the past four weeks have you gotten together socially with relatives?

ha114re How clean is the participant's building?

jx011rec Respondent’s Body Mass Index based on their reported weight and height.

hx201re Participant's self-rating of their general health. (poor [5] to excellent [1])

HX472RE First walking speed.

HX473RE Second walking speed.

hx346re Has a doctor ever told participant they have high blood sugar?

hx341re Has a doctor ever told Participant they have high blood pressure or hypertension?

hx351re Has a doctor ever told Participant they had a heart attack, coronary heart disease, angina, congestive heart failure, or other heart problems?

hx359re Does participant sometimes have pain, stiffness, or swelling in their joints?

hx348re Has a doctor ever told participant they have cancer or a malignant tumor?

hx356re Has a doctor ever told participant they had a stroke?

jx148rer Has a medical professional ever said that you have irritable bowel syndrome?

jx013rec Does Participant currently smoke cigarettes?

hc102re Century Month for beginning of current cohabiting relationship.

hc005re Century Month when current marriage began.

hc040re How close are you and your current spouse?

WLS: Siblings

ssbsex Sex of Selected Sib.

xbrdxdy Year of Sib-Respondent's Birth

OCF357 Was Father's 1957 occupation farming?

kf017j1e 1990 Major Occupation Code for first or only job in Participant's first employer job spell

kc101re Are you living in a marriage-like relationship or cohabiting?

kc102re Century Month for beginning of current cohabiting relationship

kc005re Century Month when current marriage began

kc040re How close are you and your current spouse?

pz023rer How many times, if at all, during the past four weeks have you gotten together with friends?

pz024rer How many times, if at all, during the past four weeks have you gotten together socially with relatives?

kx351re Has a doctor ever told Participant they had a heart attack, coronary heart disease, angina, congestive heart failure, or other heart problems?

kx346re Has a doctor ever told participant they have high blood sugar?

WLS: Spouses

aa002rey Year of Graduate Spouse Respondent birth.

ba002rey Year of Sibling Spouse Respondent birth.

AB044RE Was Wisconsin high school or 9th grade located in a city, town or rural area? (graduate spouse)

BB044RE Was Wisconsin high school or 9th grade located in a city, town or rural area? (sibling spouse)

**Text S2.** Participant fecal sampling instructions.


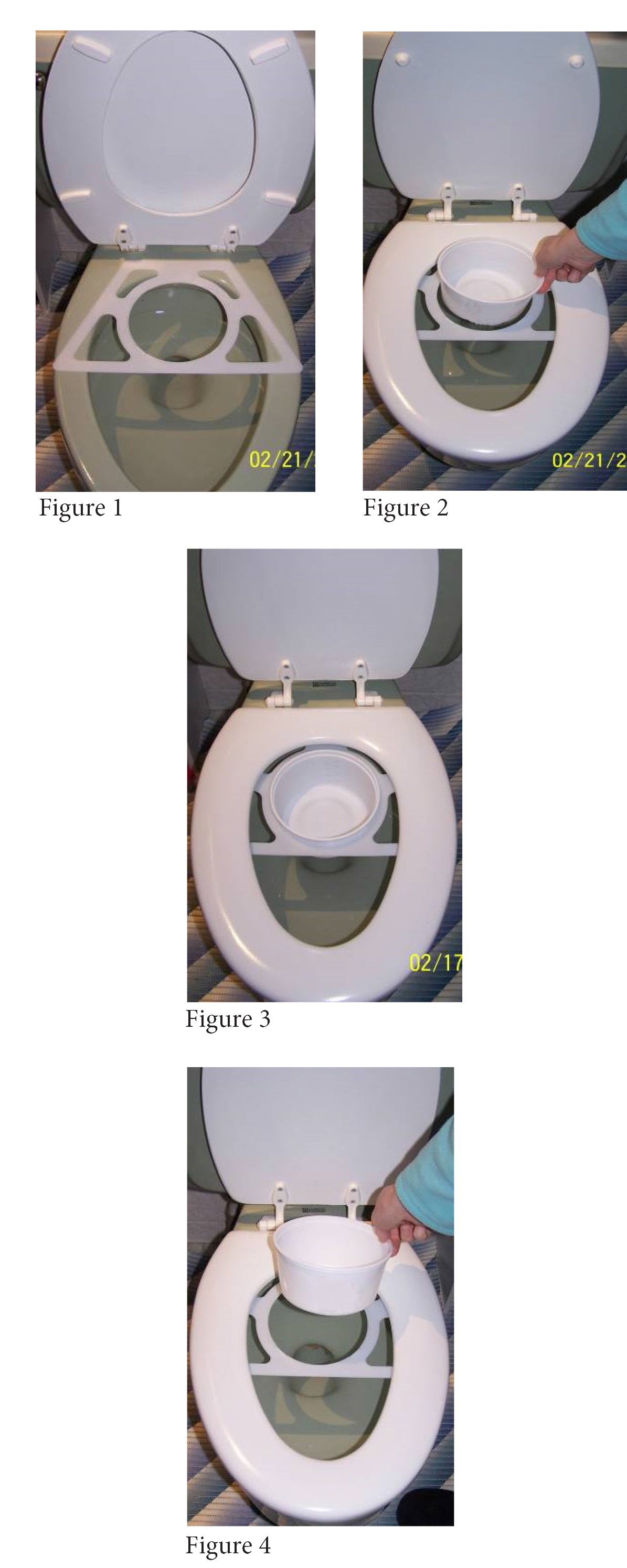


**STEP 1**

Raise the toilet seat. Place the stool collection frame on the back of the toilet bowl (see Figure 1). All four corners of the collection frame should be supported by the toilet bowl.

Place toilet seat down and set collection container in frame (see Figure 2).

**STEP 2**

Deposit your stool directly into the collection container. Do not urinate into the collection container.

**STEP 3**

After collecting your sample, remove the container from the frame (see Figure 4). Discard collection frame in trash.

**
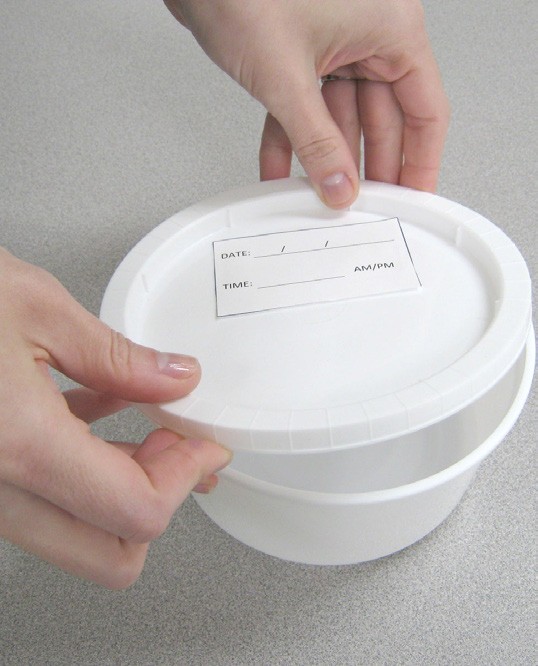

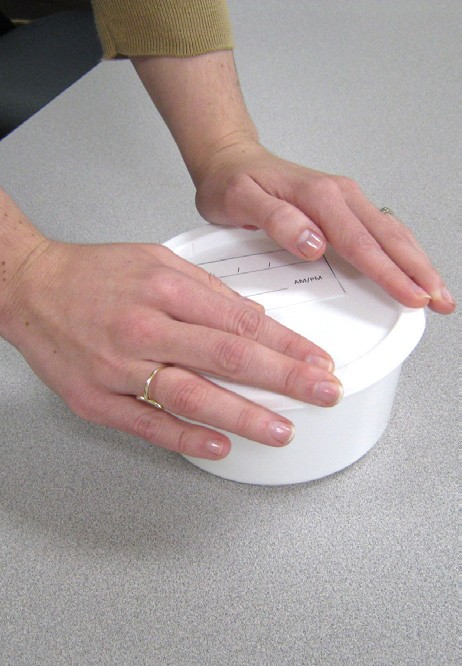
STEP 4**

Place the container on a flat surface and firmly press the lid closed (see Figures 5 and 6).

Figure 5 Figure 6


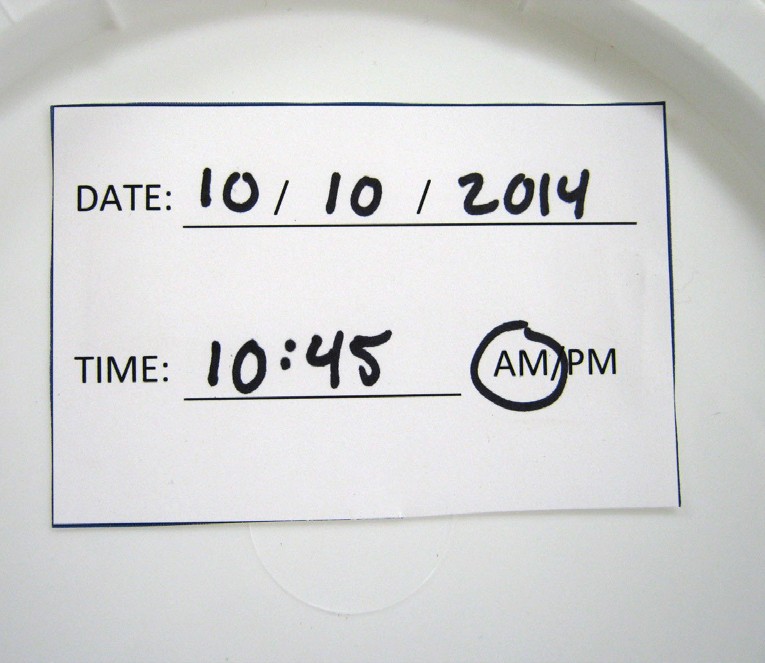
**STEP 5**

Write the date and time when the sample was produced on the provided label on the kit lid (see Figure 7).

Figure 7


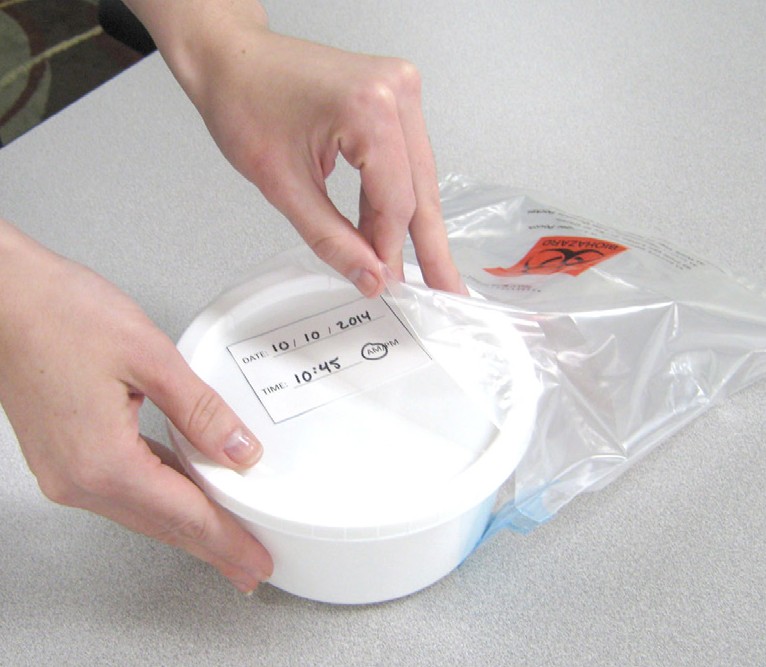
**STEP 6**

Place the closed container into the specimen bag (see Figure 8) and seal the bag by removing the blue strip to expose the adhesive.

Figure 8

**STEP 7**

Place the sample in the refrigerator immediately after it is sealed in the specimen bag. **Please, do not put your sample in the freezer.**

**STEP 8**

At your earliest convenience, please call your interviewer to schedule a pick-up.

**Dataset S1.** Fecal microbiota sequence coverage and alpha-diversity. Number of sequences and Good’s coverage for all samples both before (pre-norm) and after normalization (norm) to approximately 10,000 sequences per sample. Alpha-diversity measures including number of OTUs, Chao richness, and Shannon’s diversity were calculated from normalized data.

**Dataset S2.** Beta-diversity measures of spouse and sibling pairs. Shared OTUs and total OTUs within pairs as well as beta-diversity distances between pairs of spouses and siblings. Family IDs denote groups of related spouse and sibling pairs.

**Dataset S3.** Frequency of taxa shared within spouse and/or sibling pairs. Spouse pair counts are out of 94 and sibling counts out of 83. Individual OTUs and summed genera are shown.

**Dataset S4.** P-values of analyses of commonly shared OTUs and health outcomes across the entire dataset. Significant FDR-corrected Kruskal-Wallis P-values (*P* < 0.5) are highlighted in yellow and trends (0.5 < *P* < 0.1) in orange.
